# Neural network compensation control of magnetic levitation ball position based on fuzzy inference

**DOI:** 10.1038/s41598-022-05900-w

**Published:** 2022-02-02

**Authors:** Jiawei Tang, Zhiwen Huang, Yidan Zhu, Jianmin Zhu

**Affiliations:** 1 School of Mechanical Engineering, University of Shanghai for Science and Technology, Shanghai, 200093 China; 2 School of Mechanical Engineering, Xi’an Jiaotong University, Xi’an, 710049 China

**Keywords:** Mechanical engineering, Computer science

## Abstract

Aiming at the problem of poor transient performance of the control system caused by the control uncertainty of the undertrained neural network, a neural network compensation control method based on fuzzy inference is proposed in this paper. The method includes three control substructures: fuzzy inference block, neural network control block and basic control block. The fuzzy inference block adaptively adjusts the neural network compensation control quantity according to the control error and the error rate of change, and adds a dynamic adjustment factor to ensure the control quality at the initial stage of network learning or at the moment of signal transition. The neural network control block is composed of an identifier and a controller with the same network structure. After the identifier learns the dynamic inverse model of the controlled object online, its training parameters are dynamically copied to the controller for real-time compensation control. The basic control block uses a traditional PID controller to provide online learning samples for the neural network control block. The simulation and experimental results of the position control of the magnetic levitation ball show that the proposed method significantly reduces the overshoot and settling time of the control system without sacrificing the steady-state accuracy of neural network compensation control, and has good transient and steady-state performance and strong robustness simultaneously.

## Introduction

As a typical nonlinear and unstable system, the magnetic levitation ball system has become a common experimental platform for scholars to study control methods^1–4^. At present, the control methods of magnetic levitation ball mainly include PID control^5,6^, sliding mode control^7,8^, robust control^9,10^, adaptive control^11^, etc., but these control methods are difficult to achieve high-precision position control of magnetic levitation ball.

The neural network has adaptive and self-learning capabilities^12–14^, can accurately approximate nonlinear systems^15^. It can directly estimate the unknown nonlinear function without prior information of the closed-loop system^16^, and can improve the position control accuracy of the magnetic levitation ball^17^. Chen et al. proposed a sliding mode control method based on radial basis function (RBF) neural network, which significantly improved the control accuracy and robustness of the magnetic levitation ball control system^18^. In response to the problems in neural network training, Patan et al. proposed an adaptive iterative learning control method, which greatly improved the convergence speed and stability of neural network controller in maglev control system^19^. Fatemimoghadam et al. designed a control structure based on a recurrent neural network, which effectively improved the tracking performance of the maglev closed-loop system by introducing a time sequence relationship to predict the control quantity^20^. Jafari et al. proposed a more effective recurrent neural network training method for the identification of unknown system, which effectively reduced the tracking error of maglev system^21^. Zhu et al. used neural network identifier to establish the dynamic model between control system error and control quantity in the control loop, and dynamically copied its training parameters to the neural network feedback compensation controller, which significantly improved the steady-state accuracy of magnetic levitation ball position control^22^.

Although the above methods can effectively improve the steady-state accuracy of the magnetic levitation ball control system, the neural network controller is difficult to effectively improve the dynamic performance of the control system because the modeling accuracy of the neural network depends heavily on the amount of the training samples. Especially when tracking a reference signal with fast mutation characteristics such as step and square signals, the signal jump moment lacks sufficient sample data to effectively train the neural network, which leads to the uncertainty of the output of the neural network controller at this time. However, there are few in-depth studies on the dynamic quality of neural network control in the existing literature. Therefore, it is necessary to adopt an intelligent algorithm to adaptively adjust the output of the neural network controller at the moment of signal transition to suppress the uncertainty interference of the undertrained neural network to the control system.

As an intelligent algorithm, fuzzy inference has the advantages of clear interpretation and reliable reasoning. It is often used to solve uncertain inference problems^23–27^. Naresh Kumar et al. proposed a fault location method based on fuzzy inference for the uncertainty of faults, compared with the existing location methods, the proposed solution has been obtained higher fault location accuracy^28^. Song et al. applied fuzzy inference to fault diagnosis, and verified the effectiveness of introducing fuzzy inference through the study of uncertainty interference in mechanical structure faults^29^. In terms of uncertainty inference of control system, Tong et al. designed a fuzzy state observer for the uncertainty of the nonlinear system to estimate the unmeasured state, the results show that the method effectively improved the control Performance^30^. Based on fuzzy inference, Hu et al. used an improved genetic algorithm to optimize the fuzzy rules, which effectively improved the control effect and anti-interference performance of micro-unmanned helicopter^31^. Ko et al. introduced fuzzy inference into the traditional linear quadratic regulator (LQR) control structure, and effectively improved the power quality of the wind-hybrid power generation system by estimating the uncertain factors in the LQR control^32^. Ali proposed a control structure based on fuzzy inference, which provided an effective solution for obtaining the best agricultural indoor temperature^33^. In the electric power steering control problem, Cao et al. used fuzzy inference mechanism to control it, which significantly improved the dynamic performance of the vehicle^34^. In order to solve the active power filter current control problem under uncertainty, Hou et al. used type-2 fuzzy approach to significantly improve system performance^35^. Although these studies show that fuzzy inference has the potential to solve control uncertainty, the current literature still lacks specific research on the dynamic quality of neural network control.

In order to address the problem of poor transient performance of the control system caused by the control uncertainty of the undertrained neural network, a neural network compensation control method based on fuzzy inference is proposed for magnetic levitation ball position control in this paper, and the simulation and experimental researches are carried out. The results show that this method can improve the transient quality of neural network control system.

The main innovations of the proposed method are as follows:

1. A magnetic levitation ball position control structure based on fuzzy inference to adaptively adjust neural network control is proposed, which improves the transient performance of the control system;
2. The fuzzy inference block is designed to adaptively adjust the compensation control quantity of the neural network controller, and the dynamic adjustment factor is added to enhance the stability of the control;
3. Simulation and experimental results show that the proposed method significantly reduces the overshoot and settling time of the control system without sacrificing the steady-state accuracy.

The rest of the paper is organized as follows. “Control principle” section introduces the control principle of the proposed control structure. “Fuzzy inference block design” section describes the design of fuzzy inference block. “Results and discussion” section verifies the effectiveness of the proposed control method through simulation and experiment. “Conclusion” section is the conclusion.

## Control principle

### Control structure

**Figure 1:**
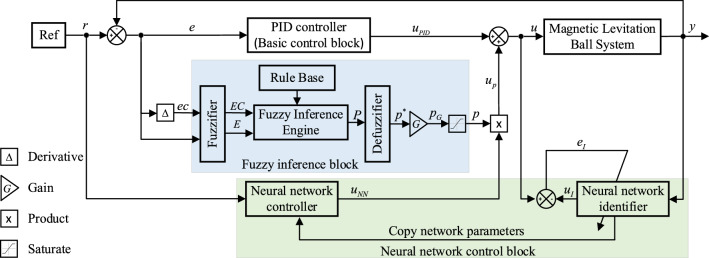
Structure diagram of neural network compensation control based on fuzzy inference.

The principle of neural network compensation control based on fuzzy inference proposed in this paper is shown in Fig. 1. Its control structure is mainly composed of three parts: fuzzy inference block, neural network control block and basic control block. The fuzzy inference block is composed of fuzzy interface, rule base, fuzzy inference engine and defuzzification interface. The neural network control block is composed of identifier and controller. The basic control block selects PID controller with good adaptability and strong robustness. The main purpose of adding the basic control block is to ensure the stability of the control system when the neural network is not fully trained or is subject to large interference.

In each control cycle, the output $y$ and input $u$ of the controlled system sampled in real time are used as the training samples of the neural network identifier, and the error between the output $u_{I}$ of the neural network identifier and the control variable $u$ is used to back propagate and update the parameters of the neural network identifier, so as to establish the dynamic inverse model of the controlled system. The parameters of the neural network identifier will be dynamically copied to the neural network controller in each control cycle. The fuzzy inference engine infers the dynamic adjustment factor $p$ according to the error $e$ and the error change rate $ec$. The control system adaptively adjusts the output of the neural network controller based on the dynamic adjustment factor. According to the expected output $r$, the neural network controller obtains the feedforward control quantity $u_{NN}$ through the feedforward calculation. After adaptive adjustment of fuzzy inference, it acts on the controlled system together with the output of the PID controller.

At the initial stage of control and the moment of tracking signal transition, the neural network lacks effective training samples. At this time, the feedforward control output by the undertrained neural network controller has strong uncertainty, and the transient performance of the control system is poor. Therefore, the fuzzy inference block is introduced to weaken the effect of the neural network controller in the insufficient training stage, and the influence of the instability of the undertrained neural network on the transient performance of control system is weakened through this adaptive adjustment mechanism.

As the control system enters a steady state, effective training samples gradually increase, the dynamics inverse model of the controlled system is more accurate, and the control error and error rate of change are getting smaller and smaller. At this time, in order to ensure the dominant role of the neural network controller in the steady state of the control system, the adaptive adjustment effect of the fuzzy inference block on the neural network controller is gradually weakening, which also ensures the steady state performance of the control system. The control structure in Fig. 1 significantly improves the transient performance of the control system while ensuring that the control system has good steady-state accuracy.

### Control algorithm

#### Calculation of control quantity

The input of the controlled system consists of the output of PID controller and the adaptively adjusted output of neural network controller. The adaptively adjusted output of neural network controller is obtained by multiplying the output of the fuzzy inference block and the output of the neural network controller.

The total control quantity of the control system is calculated as follows:

1 $$ u = u_{PID} + u_{p} $$

where $u_{PID}$ is the output of PID controller, and $u_{P}$ is the output after adaptive adjustment of neural network controller.

The basic control output $u_{PID}$ is calculated as follows:

2 $$ u_{PID} = k_{p} e(k) + k_{i} \sum\limits_{i = 0}^{k} {e(i)\Delta t + k_{d} \frac{(e(k) - e(k - 1))}{{\Delta t}}} $$

where $k_{p}$, $k_{i}$ and $k_{d}$ are the three hyperparameters of the PID controller, and $k$ represents the current sampling time.

The compensated control output $u_{p}$ is calculated as follows:

3 $$ u_{p} = p \cdot u_{NN} $$

where $u_{NN}$ is the output of the neural network controller after forward propagation, and $p$ is the output of the fuzzy inference block.

**Figure 2:**
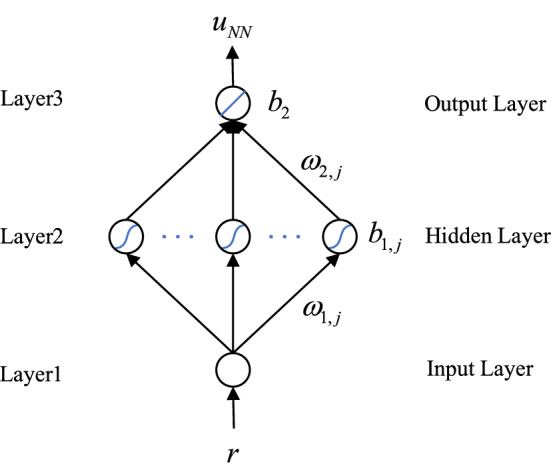
The architecture of neural network controller.

The architecture of neural network controller is shown in Fig. 2. The neural network has three layers, namely input layer, hidden layer and output layer. The number of neurons in the hidden layer is 5, the activation function is Sigmod, and the input is the target value $r$. The output of the neural network controller $u_{NN}$ is calculated as follows:

4 $$ u_{NN} = \sum\limits_{j = 1}^{n} {[\omega_{2,j} \cdot \sigma (\omega_{1,j} \cdot r + b_{1,j} )]} + b_{2} $$

where *j* = 1, 2, …, *n*, *n* represents the number of hidden layer neurons, $\omega_{1,j}$ represents the weight between the input layer and the *j*th neuron of the hidden layer, $\omega_{2,j}$ represents the weight between the output layer and the *j*th neuron of the hidden layer, $b_{1,j}$ represents the bias of the *j*th neuron in the hidden layer, $b_{2}$ represents the bias of the output layer neuron, and the symbol $\sigma$ represents the activation function.

It is worthy noted that, the parameters of the neural network controller are obtained by passing the real-time parameters of the neural network identifier. At the *K*th sampling time, the neural network identifier used the control quantity $u$ and the output quantity $y$ as a set of training samples for one training. The trained parameters will be used to calculate the output $u_{NN}$ of the neural network controller at the next sampling time.

#### Training of neural network

The identifier and the controller in the neural network control block have the same network structure, and both use a three-layer BP neural network structure. The output of the controlled system $y$ is used as the input of the neural network identifier, and the output of the neural network identifier $u_{I}$ is obtained through the forward propagation of the neural network identifier. The parameters of the neural network identifier are updated by back propagation through the loss value between $u_{I}$ and the target value $u$, and the updated parameters are used as the parameters of the neural network controller at the next time.

The training of the neural network identifier is to establish an accurate inverse model of the controlled system, and the goal is to reduce the loss value between $u_{I}$ and $u$.

The definition of the loss function is as follows:

5 $$ V = \frac{1}{2}(u - u_{I} )^{2} $$

The update formula of weight and bias in neural network is as follows:

6 $$ \omega (k + 1) = \omega (k) - \alpha \frac{\partial V}{{\partial \omega }} + \beta [\omega (k) - \omega (k - 1)] $$

7 $$ b(k + 1) = b(k) - \alpha \frac{\partial V}{{\partial b}} + \beta [b(k) - b(k - 1)] $$

where $\omega$ is the weight, $b$ is the bias, $\alpha$ is the learning rate and $\beta$ is the parameter about the momentum of the gradient. The purpose of introducing this parameter is to make the controller have a certain prediction function.

## Fuzzy inference block design

Existing research shows that adaptive neural network can effectively improve the steady-state accuracy of the control system, but due to its poor transient performance, it has not been widely used in the industry. Fuzzy inference effectively solves many uncertain problems, and is usually applied to adaptive control of unknown nonlinear systems^36^. Its principle is simple and easy to design, which can better improve the transient performance of the control system^37^. Therefore, fuzzy inference is introduced into neural network control to improve transient performance.

### Fuzzy variables and membership functions

The error $e$ and error change rate $ec$ directly reflect the state of the magnetic levitation ball^38^, so the error and error change rate are selected as the input variables of the fuzzy inference block. After the calculation of fuzzification, fuzzy inference engine and defuzzification, the $p^{*}$ used to calculate the final dynamic adjustment factor is obtained. The input variable is divided into seven fuzzy sets, namely: “Negative Big” (NB), “Negative Middle” (NM), “Negative Small” (NS), “Zero”(ZE), “Positive Small” (PS), “Positive Middle” (PM) and “Positive Big” (PB). The output variable is divided into five fuzzy sets, namely: “Small Small” (SS), “Small Big” (SB), “Middle” (M), “Big Small” (BS) and “Big Big” (BB). The value ranges of fuzzy input and output variables are shown in Table 1.

**Table 1:** Domain of fuzzy variables.

Serial number	Variable	Domain
1	$e$	(− 10, 10)
2	$ec$	(− 20, 20)
3	$p^{*}$	(0, 1]

**Figure 3:**
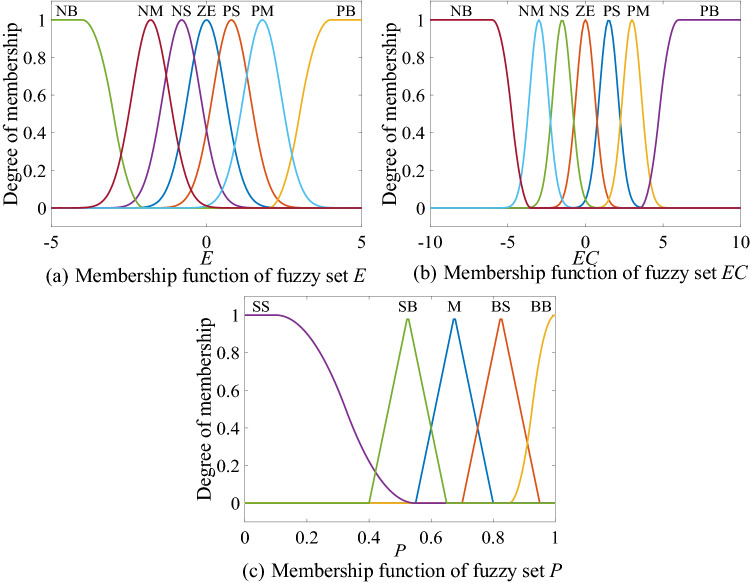
The membership functions of fuzzy inference.

In real-time control process, the drastic changes in the output of fuzzy inference will lead to the instability of the control system. The Gaussian membership function can smooth the fuzzy inference output^39^, so the fuzzy set $E$ and the fuzzy set $EC$ contain 5 Gaussian types membership function, 1 Z-type membership function and 1 S-type membership function. Triangular membership function has the advantages of simple structure and easy operation, and is widely used in real-time calculation of large-scale systems^40,41^. In order to speed up the operation speed of defuzzification, the designed fuzzy set $P$ includes 3 triangular membership functions, 1 Z-type membership function and 1 S-type membership function. The membership functions of fuzzy inference input and output are shown in Fig. 3.

### Fuzzy rules and defuzzification

Fuzzy rules are the most important part of the magnetic levitation ball control system proposed in this paper. It consists of a series of fuzzy conditional sentences. The inputs of fuzzy inference are error and change rate of error, which are transformed into fuzzy output $P$ through fuzzy rules. The fuzzy logic described in IF–THEN language is as follows:

8 $$ {\text{if}} E = A_{m} \;\;and\;\;EC = B_{n} ,\quad then\;P = C_{mn} $$

where $E$, $EC$ and $P$ are fuzzy sets defined on fuzzy input and output domains respectively.

The design principle of fuzzy rules is as follows:

1. At the initial stage of control and at the moment of tracking signal transition, there are few effective samples for training neural network, and the neural network identifier is difficult to converge quickly, so its feedforward effect is poor and the error of control system is large. At this time, the neural network identifier has not accurately established the inverse model of the controlled system, which also means that the output of the neural network controller has a strong uncertainty at this time. Therefore, in order to reduce the impact of this uncertainty on the system, we need to reduce the output of fuzzy inference, that is, increase the suppression of the output of the neural network controller. The larger the absolute value of control error, the smaller the output value of fuzzy inference.
2. When the tracking signal changes violently, the state of the magnetic levitation ball system changes obviously. The inverse model of neural network cannot converge quickly and needs a certain time to adjust the change of control system state. At this time, the absolute value of error change rate is large, so it is necessary to appropriately weaken the control function of neural network, that is, reduce the output of fuzzy inference. Therefore, the larger the absolute value of error change rate, the smaller the output value of fuzzy inference should be.
3. With the increase of sampling, the neural network inverse model is more accurate, the feedforward control effect of neural network controller is better, and the error and error change rate are small. At this time, the output value of fuzzy inference should be increased and the adjustment of the output of neural network controller should be reduced. Therefore, the lower the absolute value of the error $e$ and error change rate $ec$, the smaller the output of fuzzy inference should be.

Based on the above expert experience, using fuzzy language and fuzzy logic to transform the above criteria into a fuzzy rule table as shown in Table 2.

**Table 2:** The list of fuzzy rules.

*EC*	*E*						
	NB	NM	NS	ZE	PS	PM	PB
NB	SS	SS	SB	SB	SB	SS	SS
NM	SS	SB	M	M	M	SB	SS
NS	SS	M	M	BS	M	M	SS
ZE	SS	M	BS	BB	BS	M	SS
PS	SS	M	M	BS	M	M	SS
PM	SS	SB	M	M	M	SB	SS
PB	SS	SS	SB	SB	SB	SS	SS

As shown in Table 2, the first fuzzy rule of $P$ can be expressed as:

9 $$ R_{p1} = (u_{NB} (E) \times u_{SS} (P)) \cdot (u_{NB} (EC) \times u_{SS} (P)) $$

There are a total of 49 fuzzy rules, corresponding to 49 fuzzy relations. Taking the union operation of these fuzzy relations, the fuzzy relation formula of $P$ is as follows:

10 $$ R_{p} = R_{p1} \cup R_{p2} \cdot \cdot \cdot \cup R_{p49} = \mathop \cup \limits_{i = 1}^{49} R_{pi} $$

According to the synthesis principle of fuzzy inference, the fuzzy set corresponding to $P$ is obtained as follows:

11 $$ P = (E \times EC) \circ R_{p} $$

**Figure 4:**
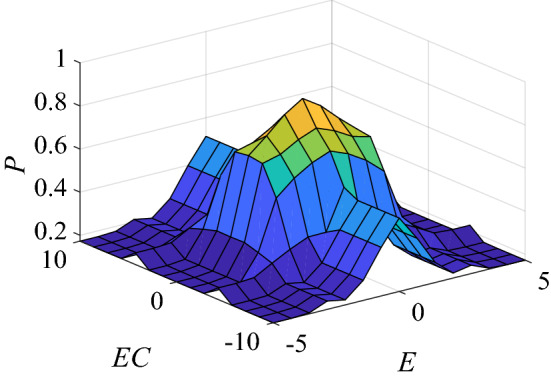
Fuzzy inference input and output surface.

The defuzzification method in this paper adopts the center of gravity method, and the input–output surface of the fuzzy inference block is shown in Fig. 4.

Fuzzy inference gets the output $p^{ * }$, $p^{ * }$ is amplified by the gain block $G$ to get $p_{G}$. When the error and error rate of change are jittered severely, the output jitter of fuzzy inference is also obvious, which will cause the control system to be disordered. In order to avoid the unstable state of the system, a saturation block is added to limit the output change rate of the fuzzy inference block. The calculation expression is as follows:

12 $$ p(k) = \left\{ {\begin{array}{*{20}l} {p_{G} (k)} \hfill & {if\;\left| {p_{G} (k) - p(k - 1)} \right| \le \xi } \hfill \\ {p(k - 1) + \xi } \hfill & {if\;p_{G} (k) - p(k - 1) > \xi } \hfill \\ {p(k - 1) - \xi } \hfill & {if\;p_{G} (k) - p(k - 1) < - \xi } \hfill \\ \end{array} } \right. $$

where $\xi$ is the change rate limit parameter of the fuzzy inference output, and the change rate of dynamic adjustment factor $p$ does not exceed $\xi$.

## Results and discussion

### Simulation results

In order to verify the effectiveness of the control method proposed in this paper, a simulation study is carried out on the magnetic levitation ball position control system. The transfer function of the magnetic levitation ball position control system is as follows^42^:

13 $$ G(s) = \frac{77.8421}{{0.0311s^{2} - 30.5250}} $$

Three different control structures are compared: PID controller, BP neural network controller + PID controller (BP + PID) and fuzzy inference + BP neural network controller + PID controller (FI + BP + PID). In addition, in order to test the effect of introducing fuzzy inference into neural network feedforward compensation control, the control parameters of PID controller used in the three control structures are the same, and the neural network structure and parameters are the same.

The tracking control targets are step signal and square signal. The simulation research will verify whether the introduction of fuzzy inference mechanism in the neural network feedforward compensation control can effectively improve the transient characteristics of the control system. The sampling time of the control system is 0.003 s, through multiple adjustments, a set of optimal PID parameters are determined, and their values are as follows: $k_{p}$ = 8, $k_{{\text{i}}}$ = 6, $k_{{\text{d}}}$ = 0.2. This paper use some values in the range of -0.1 to 0.1 to initialize the neural network parameters randomly, and the range of parameter initialization should not be too large, otherwise it may cause the instability of the control system. Hyperparameters $\alpha$ and $\beta$ have an impact on the training of neural networks. In the real-time square signal tracking control, the optimal value is determined by comparing multiple groups of different values. To search for optimal hyperparameters as wide as possible, $\alpha$ was varied from 0.006 to 0.015 in the simulation, and $\beta$ was varied from 0.86 to 0.95 in the simulation. Five simulations were performed for each hyperparameter, and the average of the overshoots in the five simulations was finally obtained. The effects of hyperparameters $\alpha$ and $\beta$ on overshoot are shown in Table 3, and the hyperparameters of the neural network are set as follows: $\alpha$ = 0.012, $\beta$ = 0.9. The gain coefficient $G$ of the fuzzy inference block is set to 1.13, and the fuzzy inference output change rate limit parameter $\xi$ is set to 0.003.

**Table 3:** The effects of hyperparameters $\alpha$ and $\beta$ on overshoot in simulation.

$\alpha$	0.006	0.007	0.008	0.009	0.010	0.011	**0.012**	0.013	0.014	0.015
Overshoot (mm)	0.210	0.206	0.194	0.165	0.169	0.151	**0.125**	0.143	0.162	0.191
$\beta$	0.86	0.87	0.88	0.89	**0.90**	0.91	0.92	0.93	0.94	0.95
Overshoot (mm)	0.206	0.189	0.156	0.131	**0.123**	0.129	0.142	0.150	0.166	0.178

Significant values are in bold.

#### Step signal tracking simulation

**Figure 5:**
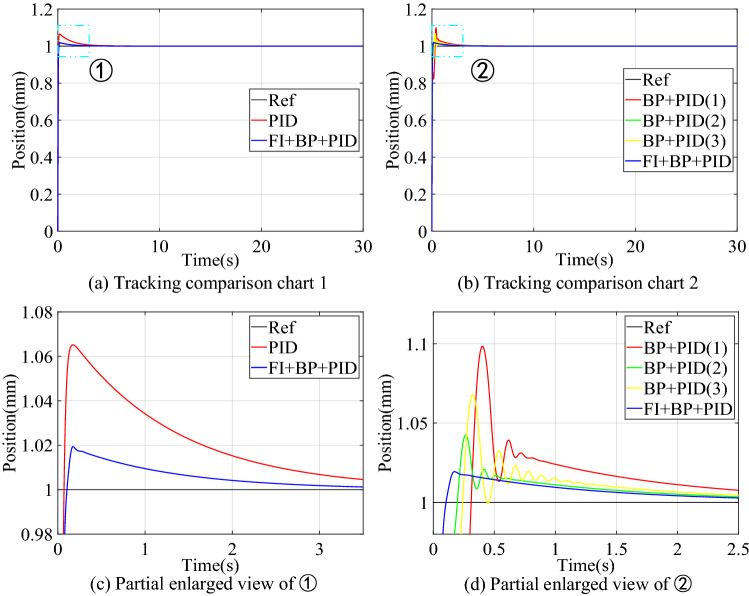
Simulation results of step signal tracking control.

The value of the step signal is 1, and the simulation result of tracking control is shown in Fig. 5. Figure 5a and b are tracking comparison diagrams, and Fig. 5c and d are partial enlarged diagrams. In order to verify the instability of the control structure of BP + PID, three simulation experiments were performed to compare it. The transient performance comparison of the three control structures is shown in Table 4.

**Table 4:** Comparison of transient performance of step signal simulation.

Control structure	Overshoot (mm)	Settling time (s)
PID	0.065	0.527
BP + PID	0.068	0.367
FI + BP + PID	0.019	0.064

The results in Fig. 5 show that the transient performance of the three simulations of BP + PID such control structure is poor and fluctuates greatly. This is because there are few training samples in the initial stage of control when tracking the step signal, which leads to the incomplete training of the neural network, and then leads to the uncertainty of the compensation output of the neural network controller. Table 4 compares the transient performance of the three structures mentioned above in the form of numbers, and the results show that the feedforward neural network compensation controller has a slight increase in overshoot and an unstable control state compared with the PID controller, but the settling time to stabilize is significantly reduced. When we introduce a fuzzy inference block to adaptively adjust the output of the neural network controller, the control system overshoot is reduced to 0.019 mm, and the settling time is shortened to 0.064 s.

**Figure 6:**
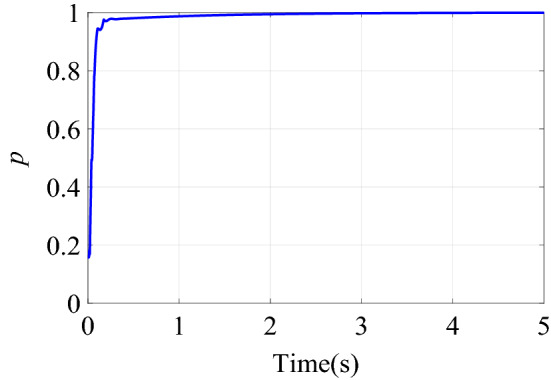
Dynamic adjustment factor $p$ of step signal tracking simulation.

Figure 6 shows the change of the dynamic adjustment factor. It can be seen form Fig. 6, in the early stage of control, due to the relatively large error and error rate of change, the fuzzy inference block adjusts the output of the neural network controller more obviously. With the online training of neural network, the error of neural network inverse model is getting smaller and smaller. The feedforward control output of the neural network controller is more accurate, and the error and error rate of the control system are also reduced. With the increase of online training samples, the restrictions of fuzzy inference block on neural network compensation control are becoming weaker and weaker. Therefore, the adjustment of the output of the neural network controller by the fuzzy inference block suppresses the uncertainty of neural network compensation control.

**Figure 7:**
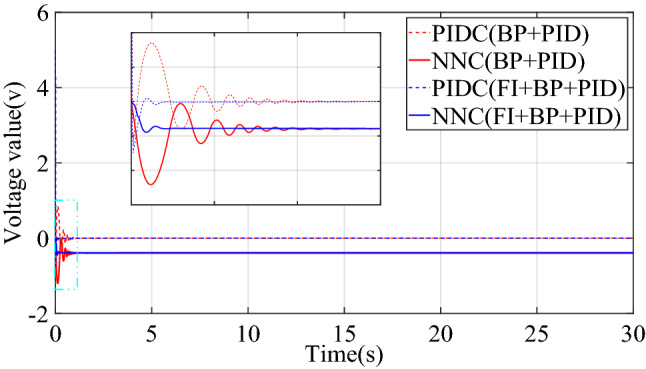
Control quantities comparison of step signal tracking simulation.

This paper compared the output voltage changes of PID controller (PIDC) and neural network controller (NNC) in the two control structures of BP + PID and FI + BP + PID. The comparison result is shown in Fig. 7, where PIDC(BP + PID) represents the output of the basic control module in the control structure of BP + PID. As shown in Fig. 7, fuzzy inference mainly plays an important role in the early stage. The introduction of fuzzy inference mechanism reduces the oscillation of the neural network controller output, thereby improving the transient characteristics of the control system. Moreover, before and after the introduction of fuzzy inference mechanism, the steady-state output of neural network controller does not change, which also ensures the steady-state performance of the control system. By comparing the control effects of the two controllers, BP + PID and FI + BP + PID, it is shown that the introduction of fuzzy inference can bring better control effect under the condition of losing some brevity.

#### Square signal tracking simulation

**Figure 8:**
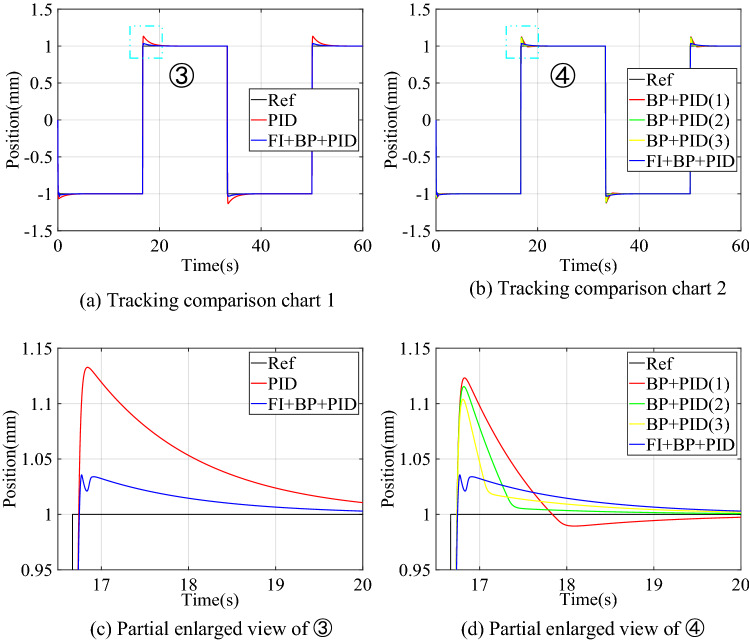
Simulation results of square signal tracking control.

The amplitude of the square signal is 1, and the frequency is 0.03. The tracking control simulation result is shown in Fig. 8. The transient performance comparison of the three control structures is shown in Table 5. The results show that the state of the control system changes drastically at the moment of the square signal transition. At this time, the neural network controller cannot output the correct control quantity due to the lack of training samples, so the transient performance of BP + PID control structure is not ideal. The overshoot of the proposed structure is reduced to 0.034 mm, and the settling time is reduced to 0.075 s. The simulation results shown in Fig. 8 and Table 5 show that the control structure of FI + BP + PID still has better transient performance in square signal tracking.

**Table 5:** Comparison of transient performance of square signal simulation.

Control structure	Overshoot (mm)	Settling time (s)
PID	0.133	1.410
PID + NNC	0.123	0.667
FI + BP + PID	0.034	0.075

**Figure 9:**
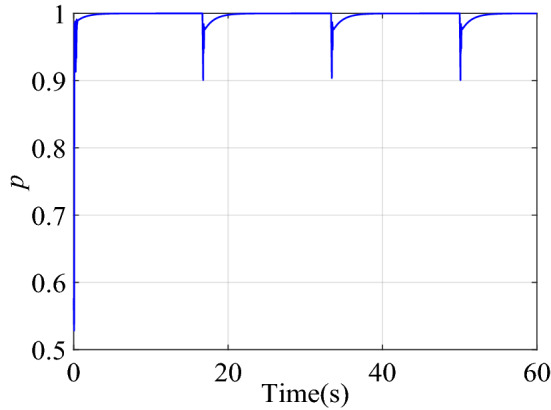
Dynamic adjustment factor $p$ of square signal tracking simulation.

**Figure 10:**
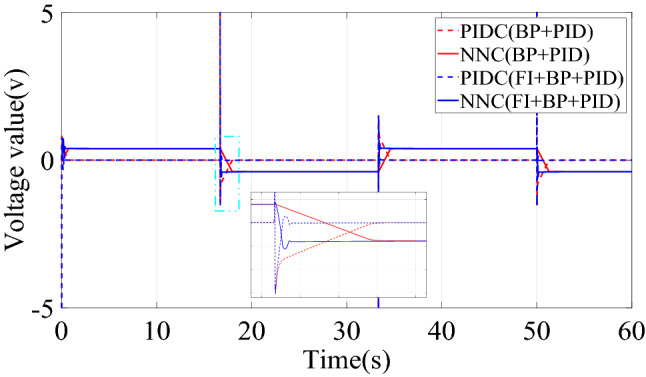
Control quantities comparison of square signal tracking simulation.

The change curve of the dynamic adjustment factor $p$ is shown in Fig. 9. Figure 9 shows that the neural network controller output needs to be adjusted mainly during the period of the signal transition. After the neural network identifier has established an accurate inverse model of the controlled system, there is no need to adjust the output of the neural network controller, and the established inverse model is directly used for feedforward compensation control. When the two control structures of BP + PID and FI + BP + PID track the square signal, the output voltage changes of PIDC and NNC are shown in Fig. 10. It can be seen from Figs. 9 and 10 that the fuzzy inference block mainly plays an obvious role in the initial stage of control and where the tracking signal changes violently. The adaptive adjustment of the output of the neural network by the fuzzy inference block reduces the influence of the uncertainty in the neural network training process on the control system.

**Figure 11:**
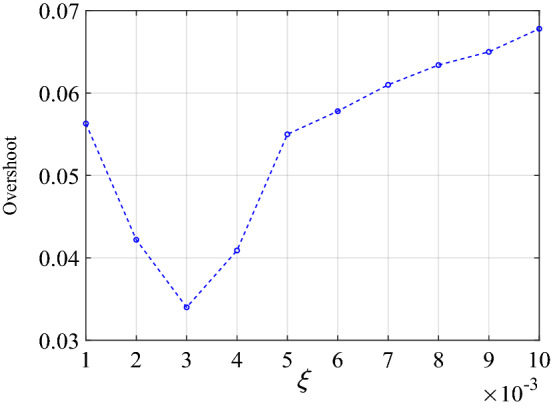
The influence of $\xi$ on simulation overshoot.

Figure 11 studies the influence of the output change rate limit parameter $\xi$ on the overshoot of the control system. The simulation found that 0.003 is the optimal value.

### Experimental results

**Figure 12:**
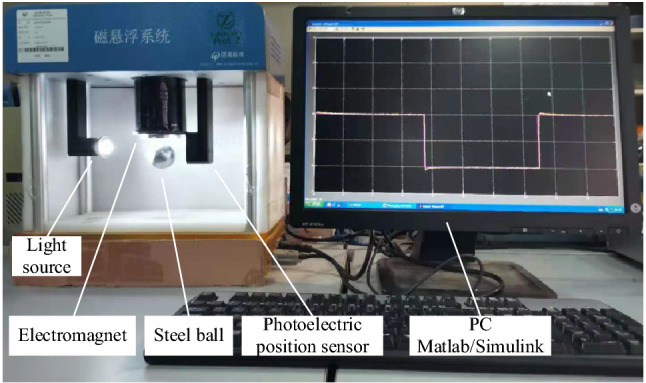
Experimental platform for position control of magnetic levitation ball.

In order to further verify the effectiveness of the proposed method in real-time control, this paper conducts an experimental study based on the magnetic levitation ball position control experimental platform. The experiment uses MATLAB / RTW software platform and PCI-1711 data acquisition card to collect the output signal of the control system. The magnetic levitation ball position control experimental platform is shown in Fig. 12.

The experiment compares the control effects of the above three control structures, and the control targets are continuous step signals and square signals.

The sampling time in the experiment is 0.003 s. A set of better PID parameters is selected through multiple experiments. The parameters of the PID controller are as follows: $k_{p}$ = 1.05, $k_{i}$ = 0.003, $k_{d}$ = 16. The experiment use values between -0.1 and 0.1 to initialize the neural network parameters randomly. In the experiment, the effects of hyperparameter $\alpha$ and $\beta$ on overshoot are shown in Table 6, and the neural network hyperparameters are set as follows: $\alpha$ = 0.015, $\beta$ = 0.04. The gain coefficient $G$ of the fuzzy inference block is set to 1.13, and the output change rate limit parameter $\xi$ is set to 0.003.

**Table 6:** The effects of hyperparameters $\alpha$ and $\beta$ on overshoot in experiment.

$\alpha$	0.011		0.012	0.013	0.014	**0.015**	0.016	0.017	0.018	0.019	0.020
Overshoot (mm)	0.660		0.515	0.423	0.342	**0.330**	0.375	0.410	0.504	0.572	0.651
$\beta$	0.036		0.037	0.038	0.039	**0.040**	0.041	0.042	0.043	0.044	0.045
Overshoot (mm)	0.584		0.494	0.418	0.349	**0.325**	0.357	0.446	0.557	0.612	0.697

Significant values are in bold.

#### Continuous step signal experiment

**Figure 13:**
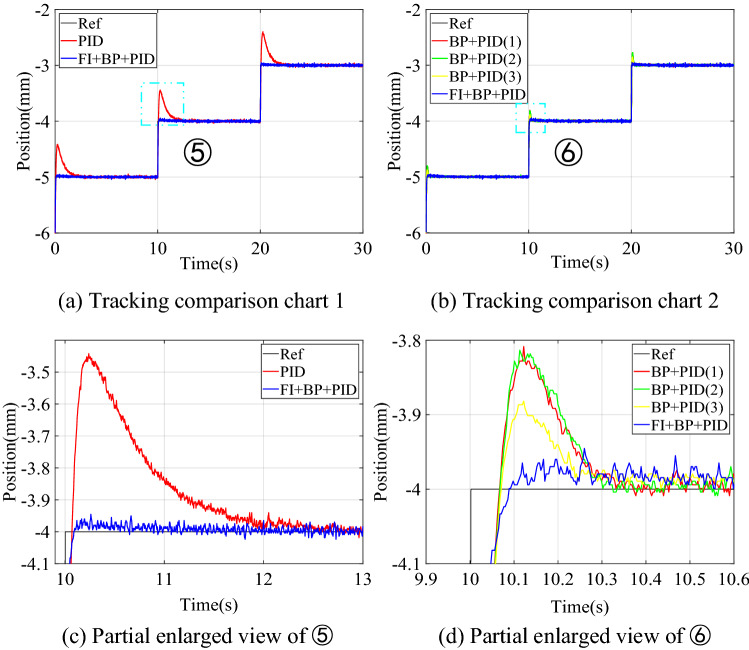
Experimental results of continuous step signal tracking control.

In the continuous step signal tracking experiment, the tracking signal stepped once every 10 s. The results of the continuous step signal tracking control experiment are shown in Fig. 13.

The performance comparison of continuous step signal experiment tracking is shown in Table 7. It can be seen from the experimental results in Fig. 13 and Table 7 that when PID controller is used alone, the overshoot of the system is 0.557 mm. After adding neural network controller, the overshoot of the system is 0.118 mm. The introduction of fuzzy inference further reduces the overshoot of the control system to 0.054 mm, and the settling time is shortened to 0.065 s. Fuzzy inference does not affect the steady-state error, and the control system still has good steady-state accuracy. The transient performance of the control system has been greatly improved, and the steady-state accuracy was also maintained.

**Table 7:** Comparison of performance of continuous step signal experiment tracking.

Control structure	Overshoot (mm)	Settling time (s)	Steady-state error (mm)
PID	0.557	1.623	[− 0.03, 0.03]
PID + NNC	0.118	0.205	[− 0.02,− 0.02]
FI + BP + PID	0.054	0.065	[− 0.02, 0.02]

**Figure 14:**
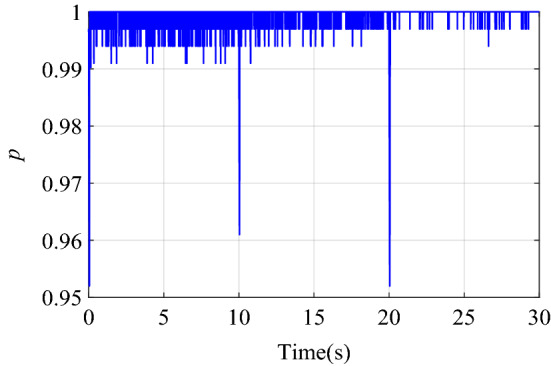
Dynamic adjustment factor $p$ of continuous step signal tracking experiment.

**Figure 15:**
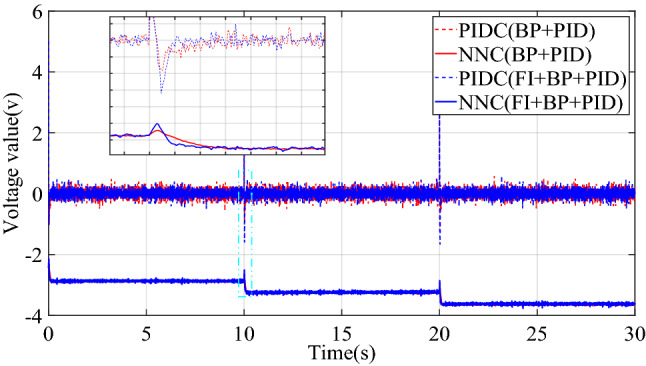
Control quantities comparison of continuous step signal tracking experiment.

Figure 14 is the change curve of the dynamic adjustment factor. The output voltage changes of PIDC and NNC in the two control structures of BP + PID and FI + BP + PID are shown in Fig. 15. From the analysis of Figs. 14 and 15, it can be seen that when the continuous step signal jumps, the fuzzy inference block increases the adjustment of the neural network controller output. With the training of the neural network, the adjustment of the fuzzy inference block is gradually weakened, which also ensures the steady-state accuracy of the control system.

#### Square signal experiment

**Figure 16:**
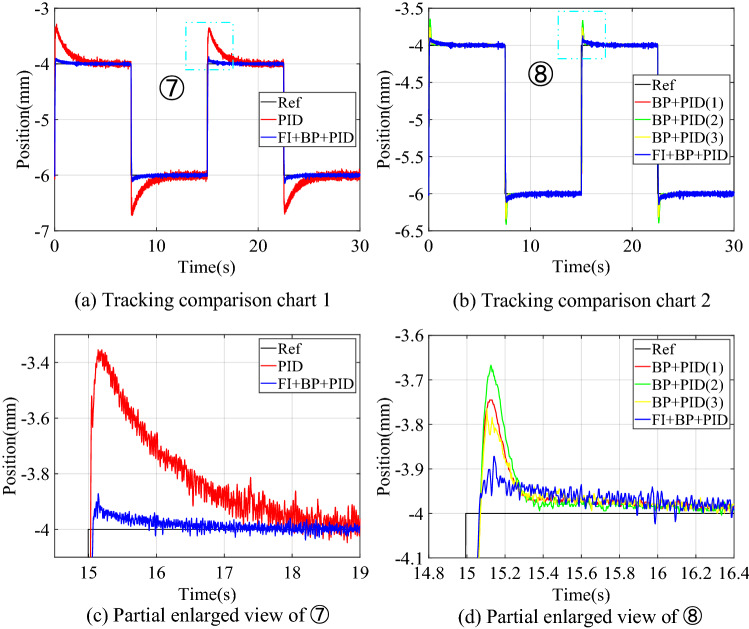
Experimental results of square signal tracking control.

The experimental results of square signal tracking control are shown in Fig. 16, and the performance comparison of square signal experimental tracking is shown in Table 8.

**Table 8:** Comparison of performance of square signal experimental tracking.

Control structure	Overshoot (mm)	Settling time (s)	Steady-state error (mm)
PID	0.715	2.132	[− 0.07, 0.07]
PID + NNC	0.318	0.215	[− 0.03, 0.03]
FI + BP + PID	0.098	0.062	[− 0.03, 0.03]

It can be seen from Fig. 16 and Table 8 that after introducing the fuzzy inference block to adaptively adjust the output of the neural network controller, the overshoot and settling time of the control system are greatly reduced without loss of steady-state accuracy. The control system obtains better transient quality.

**Figure 17:**
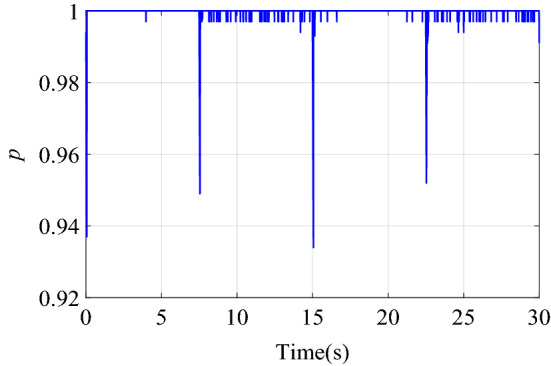
Dynamic adjustment factor $p$ of continuous square signal tracking experiment.

**Figure 18:**
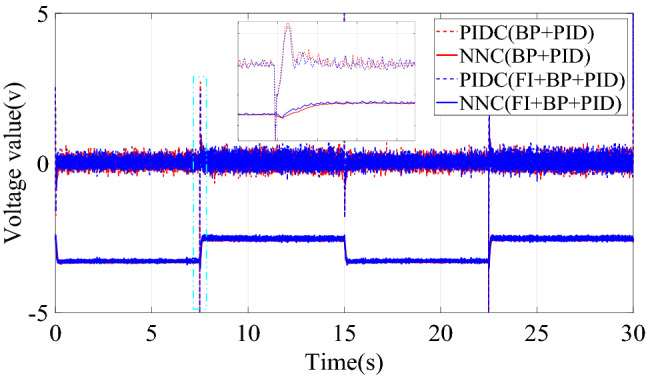
Comparison of square signal experiment tracking control quantities.

Figure 17 is the change curve of the dynamic adjustment factor. The output voltage changes of PIDC and NNC in the two control structures of BP + PID and FI + BP + PID are shown in Fig. 18. In Fig. 18, when the tracking signal jumps, the output of PID controller will increase significantly. At this time, fuzzy inference suppresses the output of the neural network controller, and the PID controller mainly ensures the stability of the control system. When the neural network training is completed, the output of the PID controller will gradually approach to zero. At this time, the stability of the system is mainly guaranteed by the neural network controller. From the analysis of Figs. 17 and 18, it can be seen that in the face of sudden changes in the tracking signal, the fuzzy inference block effectively adjusts the output of the neural network controller and ensures the steady-state accuracy.

**Figure 19:**
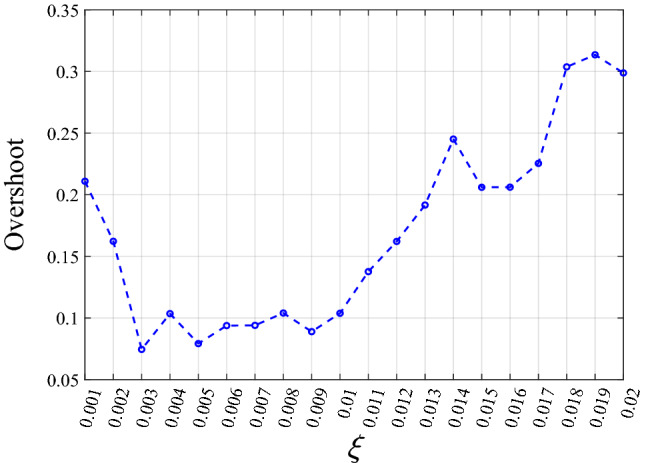
The influence of $\xi$ on experiment overshoot.

Figure 19 shows that the optimal parameter $\xi$ in the experiment is 0.003, which is consistent with the simulation results.

### Robustness experiment

**Figure 20:**
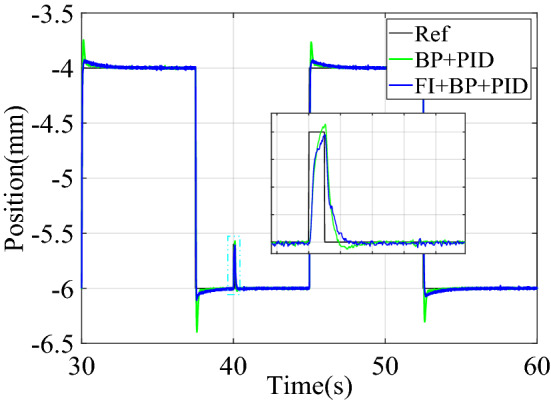
Robustness experimental results.

In order to analyze the anti-interference ability of the proposed control structure, a fixed-value interference with an amplitude of 0.6 mm and a duration of 0.1 s was added at the 40 s of the square signal tracking experiment. Figure 20 verifies the anti-interference ability of the control structure. In addition, the robustness experimental results also show that the control system can remain stable when subjected to external disturbances. It can be seen from the Fig. 20 that the control structure of FI + BP + PID can recover to a stable state faster, and the control system has better robustness.

### Discussion

At present, there are many researches on neural network controllers. Neural network control has been proven to be effective in improving the steady-state accuracy of control systems. However, in practical industrial applications, there are not many scenarios where neural networks are used. One of the main reasons is its poor transient performance. When the tracking signal has strong mutation or the control system faces some external disturbances, the undertrained neural network controller has strong uncertainty. In this manuscript, we provide an approach for the industrial application of neural networks in the control field, which effectively improves the transient performance of control systems by combining fuzzy inference with neural networks. The controller proposed in this paper is suitable for second-order systems that are not very time-critical. The disadvantage of this controller is that its control period cannot be too small, because the neural network needs to be trained in each control period. In the future, we will apply this controller to higher order systems. This is of great significance for the industrial implementation of neural network controllers.

## Conclusion

This paper takes the position control of the magnetic levitation ball as the research object, and proposes a neural network compensation control method based on fuzzy inference. This method designs a fuzzy inference mechanism based on the control error and the error rate of change to adaptively adjust the neural network compensation control quantity, and suppress the uncertain interference of the undertrained neural network to the control system. Moreover, the method adds a dynamic adjustment factor to enhance the control stability at the initial stage of network learning or at the moment of signal transition.

The simulation and experimental results show that compared with the traditional neural network compensation control under the same parameter conditions, when tracking step signal and square signal, the overshoot of the proposed method is reduced by 54.24% and 69.18% respectively, and the settling time is reduced by 68.29% and 71.16% respectively. Therefore, the proposed method significantly improves the transient quality of the control system without sacrificing the steady-state accuracy of the control system.

The proposed control method is feasible and effective. The control algorithm has reliable reasoning and stable performance, and is easy to track the reference signals with fast transition characteristics such as step and square wave.

### Consent to publish

The data used in this article have been approved by the author. The copyright to the article is transferred to Springer effective if and when the article is accepted for publication.

## Data Availability

All data generated or analysed during this study are included in this published article.
